# Cross-cultural adaptation and psychometric properties of the MMSE and MoCA questionnaires in Tanzanian Swahili for a traumatic brain injury population

**DOI:** 10.1186/s12883-019-1283-9

**Published:** 2019-04-08

**Authors:** Joao Ricardo Nickenig Vissoci, Leonardo Pestillo de Oliveira, Temitope Gafaar, Michael M. Haglund, Mark Mvungi, Blandina Theophil Mmbaga, Catherine A. Staton

**Affiliations:** 1 Duke Global Health Institute, Duke University, Durham, NC USA; 2 Duke Emergency Medicine, Duke University School of Medicine, Durham, NC 27710 USA; 3 Division of Global Neurosurgery and Neuroscience, Duke University, Durham, NC USA; 4 UniCesumar, Maringá, Paraná Brazil; 5 Duke University School of Medicine, Duke University, Durham, NC USA; 6 Kilimanjaro Christian Medical Center, Moshi, Tanzania; 7 Kilimanjaro Clinical Research Institute, Moshi, Tanzania

**Keywords:** Tanzania, Traumatic brain injury, Cognitive impairment, MoCA, MMSE, Validity, Reliability

## Abstract

**Background:**

Traumatic Brain Injury (TBI) is the most common cause of injury-related death and disability globally, and a common sequelae is cognitive impairment. Addressing post-TBI cognitive deficits is crucial because they affect rehabilitation outcomes, but doing this requires valid and reliable cognitive assessment measures. However, no such instrument has been validated in Tanzania’s TBI population. Mini-Mental State Examination (MMSE) and Montreal Cognitive Assessment (MoCA) are two commonly used instruments to measure cognitive impairment, and there have been a few studies reporting their use in post-TBI cognitive assessment. Our aim was to report the psychometric properties of the Swahili version of both scales amongst the TBI population in Tanzania.

**Methods:**

A cross-cultural adaptation committee participated in the translation and content validation process for both questionnaires. Our patient sample consisted of 192 adults with TBI who were admitted to Kilimanjaro Christian Medical Center (KCMC) in Tanzania. Confirmatory factor analysis, reliability and external validity were evaluated.

**Results:**

MoCA showed adequate factor loadings (values > 0.50 for all items except items 7 & 10) and adequate reliability (values > 0.70). Factor loadings for most of the MMSE items were below 0.5 and internal consistency was medium (< 0.7). Polychoric correlation between MMSE and MoCA was strong, positive and statistically significant (*r* = 0.68, *p* = 0.001); correlation with the cognitive subscale of FIM indicated moderately positive relationships - MMSE (*r* = 0.35, *p* = 0.001) and MoCA (*r* = 0.43, *p* = 0.001).

**Conclusions:**

With the exception of the language and memory items, MoCA is a valid and reliable instrument for cognitive impairment screening in Tanzania’s adult TBI population. On the other hand, MMSE does not appear to be an appropriate tool in this patient group, but its positive correlations with MoCA and cFIM indicate similar theoretical concepts. Both instruments require further validation studies to prove their predictive ability for screening cognitive impairment before they are considered suitable for clinical use.

**Electronic supplementary material:**

The online version of this article (10.1186/s12883-019-1283-9) contains supplementary material, which is available to authorized users.

## Introduction

Of all injuries, traumatic brain injury (TBI) is one of the most common causes of death and disability globally, and is expected to surpass many diseases as a major cause of death and disability by 2020 [1]. An estimated 10 million people are affected annually [2, 3] with over 57 million people worldwide hospitalized with one or more TBI [4]. Its sequelae include: changes in cognition, short term memory loss, attention deficit, mood disturbances, and personality changes – including impulsivity and irritability [5–16]. In particular, cognitive impairment due to TBI is a substantial source of morbidity for affected individuals, their family members, and communities at-large [17]. Deficits in attention, memory, and executive functioning are the most common neurocognitive consequences of TBI at all levels of severity [5, 18]. Especially problematic are impairments to relatively basic cognitive functions, such as attention and memory because these may cause or worsen additional deficits in executive function, communication, and other more complex processes [19]. Addressing post-TBI cognitive deficits is crucial as cognitive impairment is an important factor that affects rehabilitation outcomes [20, 21]. Hence, thorough neuropsychiatric assessment, including cognitive assessment, is essential to guiding rehabilitation efforts and appropriate medication regimens [22].

Two widely used tools for assessing cognitive function are the Mini-Mental State Examination (MMSE) and the Montreal Cognitive Assessment (MoCA). MMSE was originally developed in the United States in 1975 for dementia screening [23]. The scale assesses cognitive capacities with respect to orientation, registration, attention, recall, and language, as well as the ability to follow verbal and written commands [23]. It is probably the most popular measure to screen for cognitive impairment and has been culturally validated in many countries. While the scale is helpful in approximating gross cognitive ability, there are concerns that it does not take into account mental elasticity and working memory, [24] and that it has ceiling effects when administered to individuals with high educational circumstances [25]. The MoCA, developed in 2005, is another widely used measure of cognitive function [26]. It comprises 10 items, with varying degrees of difficulty, which assess 6 cognitive domains: executive functioning; visuospatial abilities; short-term memory; language; attention, concentration, and working memory; and temporal and spatial orientation [26]. Values for the for MMSE and MoCA are similar - 0 to 30 range, with higher scores indicating better cognitive functioning. Similar to MMSE, MoCA is also used for evaluating cognitive impairment in early dementia, but it has been shown to have greater sensitivity than MMSE when screening for mild cognitive impairment (MCI), mild dementia, and cognitive impairment resulting from stroke [27].

Specific to TBI, both MoCA and MMSE have been used to measure patient-reported post-TBI cognitive outcomes [20, 28, 29]. There have been a few studies that have evaluated the use of MoCA in these patients; Wong et al. validated the scale in TBI patients with intracranial hemorrhage in Hong Kong [29] and Kumar et al. tested the reliability of MoCA in screening for mild TBI. Zhang et al. reported on the sensitivity of MMSE in screening for post-TBI cognitive impairment. However, to our knowledge, there have been no studies reporting the construct validity and reliability of MMSE in TBI patients.

At KCMC, a regional referral hospital in northern Tanzania, TBI is the leading cause of injury-related death and disability and it represents ~ 6% of all emergency department visits [30]. Post-TBI cognitive impairment is often assessed using MMSE and MoCA since both scales are short, easy to administer, and feasible to implement as cognitive screeners. However, to date, neither tool has been psychometrically tested in Tanzania - or in its TBI population. The importance of doing this cannot be emphasized enough. For instance, due to the fact that both tools were developed in English, a recurring issue when translating these scales is that the prompts involving language abilities have had to be adapted to reflect culturally appropriate constructs. For example, the MMSE construct, “No ifs, ands, or buts” has often been replaced as no direct translation for this phrase exists in many languages [31, 32]. The phrase “Close your eyes” which is one of the command prompts in MMSE, means death in Chinese in certain contexts; so, some researchers have changed the prompt to “Raise your hands” [31, 32].

Despite the current use of MMSE and MoCA at KCMC, the Swahili versions of these scales - or any other scale - have not been validated to screen for post-TBI cognitive impairment in Tanzania, where Swahili is the primary language. This represents a deficit in the capacity to appropriately diagnose and treat post-TBI cognitive morbidity. Therefore, the aims of this study were to (a) develop the first systematic translation and adaptation of MMSE and MoCA in Tanzanian Swahili and (b) to analyse the psychometric properties of the scales in Tanzanian TBI patients, including evidence of reliability, construct validity, and external validity.

## Methods

### Study design and setting

Moshi, a municipality in the Kilimanjaro region of Northern Tanzania with a population of 184,292, is home to Kilimanjaro Christian Medical Centre (KCMC), the third largest hospital in the country and the referral hospital for northwestern Tanzania [33].

### Participants

Patients who met the inclusion criteria – being older than 18 years of age, seeking acute care for a TBI of any severity, being admitted for continued care, being able to speak and understand Swahili, being able to answer questions adequately and providing consent to participate before discharge – were approached for enrollment into this study. After going through the informed consent process, participants were enrolled in the KCMC TBI registry prior to hospital discharge. Patients responded to the Tanzanian versions of the MMSE and the MoCA at the bedside along with a longer set of mental health and functioning interview questions. Quality control for the data collected and entered was performed by the principal investigator. Questionnaire responses were gathered and stored using REDCap [34]. Further details about the KCMC TBI registry methodology can be found elsewhere [30].

### Translation and adaptations

The translation and cross-cultural adaptation committee was constituted of five bilingual researchers or research nurses who voluntarily participated in the translation, adaptation and content validation of both instruments. With the adapted translated version, we piloted the instrument with a convenience sample of 20 adult paticipants from Tanzania. The pilot test allowed us to verify the quality and the questions, and the content and language coherence of the instrument [35].

As suggested by the WHO for health outcomes translation [36], both instruments underwent our independent back translation protocol: i) native bilingual Swahili translators were hired to independently translate MMSE and MoCA into Swahili; ii) then bilingual language translators were hired to back-translate the Swahili versions to English; iii) the English back-translations were then compared with the original versions of the instruments and inconsistencies were checked by two independent bilingual researchers; and iv) the last step was to evaluate for issues with semantics and make adjustments as deemed appropriate by the researchers. Swahili and English versions of both scales are included as additional files with the current publication (Additional files 1, 2, 3 and 4).

To analyze the theoretical and content evaluation of the translated instruments, a set of focus group discussions were conducted with expert judges. This served to verify: (a) the practical relevance and (b) language clarity of the translated instruments, and (c) the theoretical coherence of the items. The experts’ opinions were discussed collectively in the focus groups sessions to address discordance and improve translation quality.

### Data analysis

Patient characteristics were reported using descriptive statistics (mean, standard deviations, medians, interquartile range and frequency distributions). All analyses were performed using the R Language for Statistical Computing software [37].

### Internal validity

To verify the construct validity of MMSE and MoCA, Confirmatory Factor Analysis (CFA) was employed, based on the prior literature looking at the factor structure in different populations. CFA is a statistical technique that is used to evaluate the appropriateness of a measurement model that is derived from prior empirical research and/or theory [38]. For both of these scales, previous studies that have analyzed the construct validities have differed in the reported factor structures. For MMSE, some studies have reported two factors [39–41]; or five factors [42]. Some studies have reported MoCA with 7 factors [43]; 6 factors [26, 44]; or 2 factors [45]. Since the purpose of this study is to evaluate MMSE and MoCA as cognitive screening tools, the scales were analyzed unidimensionally as has been done in some prior studies - Jones & Gallo et al. for MMSE [46]; Freitas et al. and Janelidze et al. for MoCA [44, 47].

Confirmatory Factor Analysis model adequacy was tested using commonly accepted indices intended to evaluate model fit [38, 48]. Weighted Least Square Means and Variance Adjusted (WLSMV). The following fit indices were used to test model adjustment: Chi-square (X^2^ and *p*-value), Root Mean Square Error of Approximation (RMSEA < 0.08, CI 95%), Tucker-Lewis index (TLI > 0.95), and Comparative Fit Index (CFI > 0.95). We also calculated the Average Variance Extracted (AVE) with values greater than 0.50 were considered satisfactory indicators of construct validity [49].

### Reliability

In order to test the internal consistency of the items, the Cronbach’s alpha, Omega and Composite Reliability analysis were performed for both instruments, considering that coefficients above 0.7 are acceptable [50].

### External validity

We tested external validity in two ways.

i. By correlating MMSE and MoCA with each other as was done in previous studies, which showed a high level of correlation between both scales [43, 51–55].
ii. By correlating MMSE and MoCA with the cognitive subscale of Functional Independence Measure (cFIM). FIM is a widely-used scale designed to determine an individual’s level of disability, as reflected by the need for assistance [56]. Higher FIM scores indicate higher levels of independence, and the scale can be divided into the motor subscale and the cognitive subscale. Prior studies have shown positive correlations (moderate to strong) between MMSE, MoCA and cFIM [21, 55, 57]; so we also administered FIM to our study sample.

Our hypothesis was that the Swahili, adapted versions of MMSE and MoCA questionnaires would correlate positively with each other and with the cognitive subscale of FIM, confirming the instruments’ ability to behave as expected in relation to their theoretical concepts.

## Results

Study participants included 192 adults, all of whom were part of KCMC’s TBI patient registry and post-hospitalization cohort study. Most of the participants were male (82.8%) and overall had an average age of 33.87 (SD = 13.32) years old; this is consistent with previously reported demographic information on TBI patients at KCMC [30]. In relation to injury, most patients (91%) had a mild TBI severity, with a Glasgow Coma Score of 13–15. Full demographic and health status characteristics of the study participants are presented in Table 1. Descriptive analysis showed that participants endorsed to all the response possibilities for each of the 11 items of MMSE and the 10 items of MoCA.

**Table 1:** Baseline characteristics of the validation sample

VARIABLES	
Age (years), Mean (SD)	33.87 (13.32)
Household size, Mean (SD)	4.43 (2.48)
Monthly personal income, USD, Median (IQR)	$67.11 (26.8;134.2)
Monthly family income, USD, Median (IQR)	$98.4 (44.7;156.6)
Male, N (%)	159 (82.8)
Married, N (%)	104 (54.7)
Occupation, N (%)	
Business	44 (21.7)
Farming	41 (22.3)
Skilled worker	23 (12.5)
Salaried worker	67 (36.4)
Other	13 (7.1)
Education, N (%)	
Some primary education	112 (59.3)
Some secondary education	44 (23.3)
Some university education	33 (17.5)
Severity of Injury	
Mild Traumatic Brain Injury (Glasgow Coma Score 13–15)	91%

### Internal validity & reliability

**Fig. 1:**
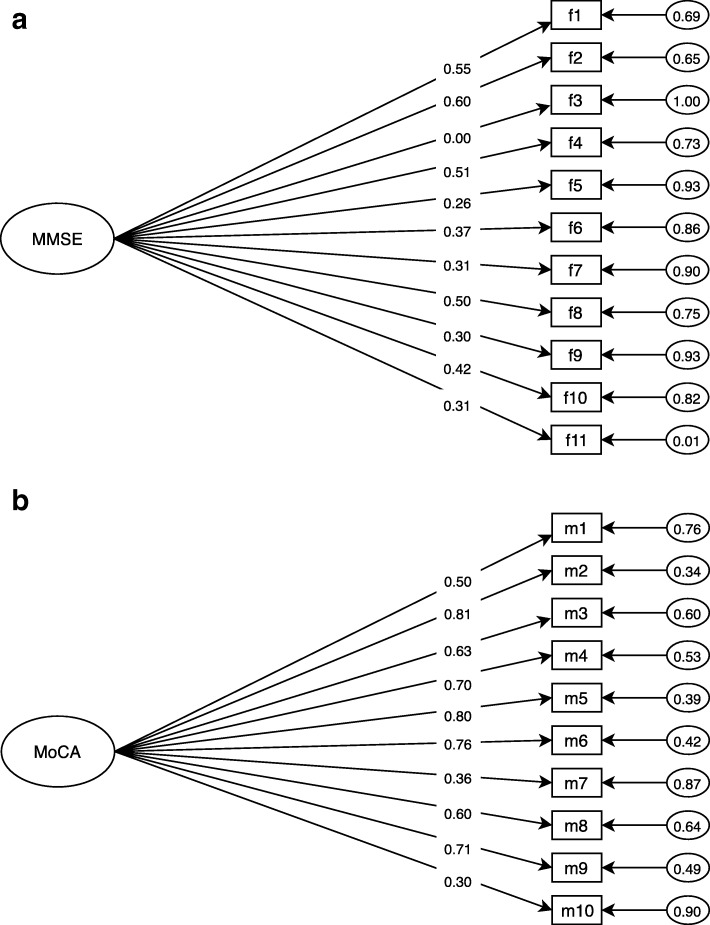
Confirmatory factor analysis diagrams, factor loadings for MMSE and MoCA. Numbers on the left of the items indicate the factor loadings while numbers on the right indicate error terms

Using CFA, both scales were analyzed uni-dimensionally, and model adequacy was tested. The fit indices indicated that a unidimensional model was adequate for MoCA but less so for MMSE (Table 2). Factor loadings were below 0.5 for 7 of the 11 items in MMSE (items 3, 5, 6, 7, 9, 10, & 11), while the rest were adequate (Fig. 1); AVE for the scale was 0.16 (Table 2). Regarding MMSE’s internal consistency, medium scores were found (below 0.7) for all the tested reliability measures (Table 2). The MoCA questionnaire showed adequate factor loadings (above 0.50) for all items, except for items 7 & 10 (Fig. 1); AVE was 0.40 (Table 2). MoCA’s internal consistency was adequate with scores above 0.70 for all reliability measures tested (Table 2).

**Table 2:** Psychometric properties for content and construct validity

	MMSE	MoCA
Reliability		
Cronbach’s Alpha	0.63 (0.60;0.66)	0.78 (0.73;0.80)
KMO	0.65	0.80
Composite Reliability	0.64	0.86
CFA		
X^2^ (Df) / *p-*value	66.27 (33) / 0.001	46.80 (45) / 0.08
RMSEA (CI 95%)	0.06 (0.04;0.08)	0.04 (0.00;0.07)
TLI	0.81	0.98
CFI	0.85	0.98
Factor loadings range	0.00–0.60	0.30–0.81
Average extracted variance	0.16	0.4

*KMO* Kaiser-Meyer-Olkin coefficient, *CFA* Confirmatory Factor Analysis, *X*^*2*^ Chi-Square, *Df* Degree of Freedom, *RMSEA* Root Mean Square Error of Approximation, *TLI* Tucker-Lewis Index, *CFI* Comparative Fit Index

Invariance analysis was performed for MoCA - the subjects were divided into 2 groups based on education level (‘some primary education’ and ‘more than primary education’). Overall, MoCA showed evidence of configural effects and intercept invariance in relation to education level. However, when the analysis was performed using the factor means model, the scale showed variance with respect to education. Specifically, for the items m7 (language) and m10 (memory), participants with some primary education performed worse than participants who had more than primary-level education. This variance was not observed when the configural model analysis was performed. Invariance analysis was not performed for the MMSE because the values of fit and its factor loadings indicated model inadequacy (Table 2).

### External validity

**Fig. 2:**
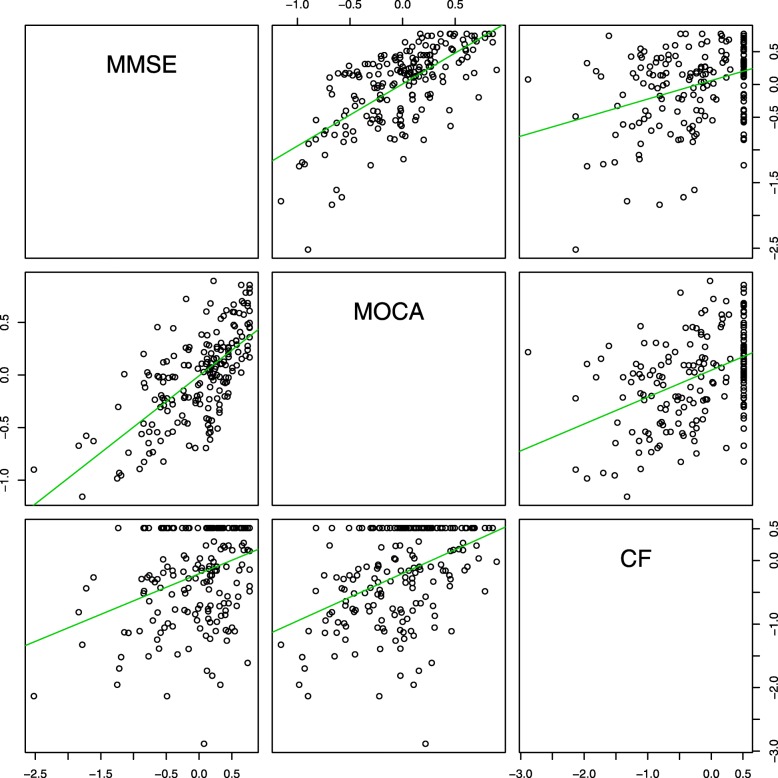
Scatterplot matrix: Correlation between MMSE, MoCA, and cFIM. Axes represent factor scores

MMSE showed a strong positive polychoric correlation (> 0.5) with MoCA (*r* = 0.68, *p* = 0.001) (Table 3, Fig. 2). When correlated with cFIM, both scales demonstrated moderately positive relationships - MMSE (*r* = 0.35 *p* = 0.001) and MoCA (*r* = 0.43, *p* = 0.001). Table 3 contains the correlation coefficients between MMSE, MoCA and cFIM - the subscale aggregate and its individual items. With the exception of two items - comprehension and memory - which were more positively correlated with MMSE, the items in cFIM had higher positive coefficients when correlated with MoCA than when correlated with MMSE. This confirms the instruments’ ability to behave as expected in relation to the theoretical concept.

**Table 3:** Correlation between MMSE, MoCA and cFIM

	MMSE (R)	MOCA (R)
MMSE	1	0.68
MOCA	0.68	1
cFIM	0.35	0.43
cF17- Expression	0.34	0.38
cF18- Comprehension	0.46	0.43
cF19 - Reading	0.43	0.57
cF20 - Writing	0.41	0.49
cF21 - Speech intelligibility	0.33	0.47
cF22 - Social Interaction	0.31	0.42
cF23 - Emotional Status	0.27	0.31
cF24 - Adjustment to limitations	0.26	0.37
cF25 - Use of leisure time	0.24	0.41
cF26 - Problem Solving	0.25	0.35
cF27 - Memory	0.36	0.26
cF28 - Orientation	0.28	0.33
cF29 - Concentration	0.37	0.41
cF30 - Safety awareness	0.34	0.36

## Discussion

The purpose of this study was to evaluate the psychometric properties of the Tanzanian Swahili versions of MMSE and MoCA as screening tools for post-TBI cognitive impairment in the country. This is the first study to report on the scales’ properties in Tanzania, and on the internal consistency and construct validity of MMSE in TBI patients. The Tanzanian version of MoCA showed satisfactory psychometric properties, with adequate reliability and content and construct validity (except for two items). MMSE, on the other hand, performed poorly; although its content validity was adequate, its reliability scores were medium, and factor loadings for most of its items were unsatisfactory. Pertaining to external validity, the significantly high level of positive correlation between MMSE and MoCA is consistent with previous literature [43, 51–54] and indicates that on a gross level, both of these instruments measure similar theoretical concepts with regards to cognitive deficits. In addition, our study found a positive correlation between MMSE, MoCA and cognitive subscale of FIM - similar to what has been reported in the literature [21, 55, 57] - providing further evidence of the external validity of both scales.

Regarding factor structure, MoCA was originally designed to have 6 dimensions: executive functioning; visuospatial abilities; short-term memory; language; attention, concentration, and working memory; and temporal and spatial orientation [26]. Exploratory analysis of MMSE demonstrated 5 robust dimensions: concentration; language/praxis; orientation; attention; and memory [42]. Functionally, however, both the MMSE and the MOCA are usually scored as a unidimensional construct, with single total score providing a global assessment of cognition [58]. Similar to prior studies that have found the MoCA to be a good MCI screening tool [44, 47], our internal structure analysis of the Swahili version of MoCA found the unidimensional model to demonstrate excellent fit. On the other hand, fitness indices indicated inadequacy in utilizing the Swahili MMSE in a unidimensional fashion.

### MoCA and TBI

Prior to this study, MoCA was validated in TBI patients with intracranial hemorrhage in Hong Kong [29]. Kumar et al. tested its reliability in screening for mild TBI and found an 87.9% sensitivity and a 66.7% specificity for detecting cognitive impairment. Therefore, prior literature recommend that the MoCA could be an effective tool to guide rehabilitation and treatment efforts [59]. Our results support these previous results; we found that the Tanzanian MoCA demonstrates adequate psychometrics in TBI patients.

With the exception of two items, the Tanzanian MoCA showed good content and construct validity. This was consistent with prior MoCA validation studies in Egypt, China, Chile, Japan, Colombia, Sri Lanka, Korea, and Brazil [43, 51–54, 60–62]. Regarding the two items (language and memory) that had unsatisfactory factor loadings, invariance analysis indicates that the education level of subjects must be taken into account and highlights the importance of analysing the scale results at an aggregate level and, also, at an individual item level. The reliability scores for each factor met internal consistency criteria - equal to or higher than 0.70 [49, 63] - and were also consistent with the existing literature [43, 51–54, 60–62]. However, given that the Cronbach’s alpha has been criticized in the psychometrics literature, we also calculated alternative reliability scores (Composite Reliability and Omega coefficients). Both scores have shown to be consistent with reduced bias [64]. In our resulrs, all reliability coefficients (Alpha, Composite Reliability, and Omega) confirmed satisfactory reliability of the Swahili version of MoCA [64].

There have been a few studies that have validated post-TBI cognitive assessment tools – these include Brain Injury Alert screening tool (BI Alert) [65] and Immediate Post Concussion Assessment and Cognitive Testing (ImPACT). However, unlike MoCA, which is widely used and has been translated and validated in many languages, these tools are not as widely available and limited evidence exists on their generalizability to other cultures or population subsets. External validity was assessed by comparing the performance of MoCA to MMSE and cFIM, but there is no established gold standard tool for measuring cognitive impairment in Tanzania. So, even though our results preliminarily verify the cross-cultural adaptation and validation of MoCA amongst Tanzania’s TBI patient population, more research is needed to establish its performance when compared with other measures of post-TBI cognitive impairment.

### MMSE and TBI

Despite the lack of prior validation studies of MMSE in Tanzania, it is often used to screen for cognitive deficits in health institutions. This is the first study to report on the psychometric properties of the Tanzanian Swahili version of MMSE, as well as the first study to report on its internal consistency and construct validity in post-TBI patients. Our results indicate that, even though the scale has been used globally and in Tanzania to monitor TBI symptoms [28, 66–70], the Swahili version of MMSE is not a good cognitive impairment screening for this population. The scale has also been found to be have less sensitivity in screening for post-TBI cognitive impairment than MoCA [20]. However, as stated earlier, MMSE’s strong correlation with MoCA, and its moderate correlation with cFIM, provide evidence that these scales evaluate similar concepts. MMSE’s poor psychometric properties indicate scale instability and high measurement error. As such, further adaptation studies and exploratory factor analyses may be appropriate to investigate how the Tanzanian Swahili version of MMSE could be adjusted and improved for post-TBI cognitive evaluation.

## Limitations

Results of this study should be taken in the context of its limitations. One limitation with this study is related to its sample, being that only TBI patients were included; however, this is also a strength of this study. Even though validation studies are usually conducted with more general populations, there is a need to evaluate the psychometric properties of these instruments in high-risk patient samples such as ours - TBI is the leading cause of death and disability due to injury at KCMC. Our results demonstrate that MoCA can provide relevant diagnostic value to help healthcare professionals in comprehending acquired cognitive deficits among TBI patients. Nevertheless, this sample is unlikely to be representative of the entire adult Tanzanian population, so further studies are warranted to evaluate the psychometric properties of the Swahili versions of the tests in the general Tanzanian population. Secondly, TBI disproportionately affects young males, in Tanzania and globally, and this is reflected in our imbalanced sample. Performances on MMSE and MoCA scales are often associated with certain demographic factors, which is the reason validation studies such as ours are typically required. However, the limited variability in our study sample means we were not able to test the influence of gender and age. Thus, applying age- and gender-based comparisons should be considered with caution.

Another factor to be considered is that m10, the one of the MoCA items that measures memory, had an inadequate factor loading, and memory is one of the most commonly impaired domains in TBI [17]. A possible explanation is the fact that the item was shown to be influenced by the education level of subjects and majority of our TBI patient sample only had “some primary education”. Another potential study limitation is the usage of MMSE in this context. While we included the MMSE due to its widespread use in post-TBI cognitive assessments, some prior literature suggests a neuroanatomical discrepancy between regions affected by TBI and those assessed by the MMSE [71]. Ultimately, our data suggest challenges with using the scale for post-TBI cognitive assessment in our setting but we can’t predict the cause of those challenges. Finally, we could not include criterion validity estimates in our study, which denotes a potential limitation to the clinical interpretation of our results. We were unable to identify current valid and reliable clinical measures for cognitive functioning in the Tanzanian culture because we were unable to include a neurologist’s assessment of our participants cognitive functioning to serve as the gold standard to allow us to calculate sensitivity and specificity and cutoff points of MoCA. Unfortunately, there is a gap in healthcare capacity related to neurology and psychiatry in Tanzania to support adequate cognitive screening, or to train healthcare providers to provide screening. As a step toward increasing capacity to assess for cognitive impairment, the current study aimed to evaluate the translation and adaptation of MMSE and MoCA to Swahili. Given that MoCA has been shown to be psychometrically adequate, we suggest that its criterion validity should be the subject of future work.

## Conclusion

Cognitive impairment is a major cause of TBI-related disabilities and an important factor that affects rehabilitation outcomes. Yet, there have been a limited number of studies that have evaluated the psychometric properties of MMSE and MoCA, two widely used cognitive screening tools in TBI patients in sub-Saharan Africa and around the globe. This is an initial report on the reliability and validity of these scales in a Tanzanian TBI patient sample. With the exception of the language and memory items, which are influenced by education level, MoCA is a valid and reliable instrument to screen for cognitive impairment in this population. MMSE does not appear to be a good cognitive screening tool in this patient sample; however, its positive correlation with MoCA and cFIM indicate similar theoretical concepts. This cross-cultural validation of MoCA further develops the capacity to screen for, measure, and treat disorders of cognitive functioning in Tanzania’s TBI patients, as well as allowing for more evidence-based practices and advances in research and policy development.

## Additional files

Additional file 1: Tanzanian Swahili version of MMSE. (DOCX 25 kb)

Additional file 2: Tanzanian Swahili version of MoCA. (DOCX 130 kb)

Additional file 3: English version of MMSE. (DOCX 21 kb)

Additional file 4: English version of MoCA. (DOCX 130 kb)

## Abbreviations

CFA: Confirmatory Factor Analysis
FIM/cFIM: Functional Independence Measure/Cognitive subscale of Functional Independence Measure
KCMC: Kilimanjaro Christian Medical Center
LMIC: Low-and Middle-Income Country
MCI: Mild cognitive impairment
MMSE: Mini-Mental State Examination
MoCA: Montreal Cognitive Assessment
TBI: Traumatic brain injury
WHO: World Health Organization
