# Supplementary material for: Cross-cultural adaptation and psychometric properties of the MMSE and MoCA questionnaires in Tanzanian Swahili for a traumatic brain injury population
Source: BMC Neurol. 2019 Apr 8;19:57. doi: 10.1186/s12883-019-1283-9 (PMC6454609; doi:10.1186/s12883-019-1283-9)

**SWAHILI TRANSLATION**

**Mini-Mental State Exam (MMSE)**

*Katika kipengere kinachofuata,nitakuuliza maswali na kutaka wewe kufanya vitu rahisi ili kujua jinsi gani ubongo wako unafanya kazi.Wakati mwingine ni ngumu,usihofu.Jibu maswali kadiri unavyoweza.Una sekunde 10 kwa maswali yote.(Mhojaji, baada ya kuuliza maswali,tiki kama mgonjwa amejibu swali kwa usahihi.kama sahihi,chagua sahihi kwa kushuka au chagua jibu linaloendana kwa kushuka)*

**KUPIMA MMSE (Mini-Mental State Exam)**

**F1**.

1. Huu ni mwaka gani? Jibu si sahihi | Jibu sahihi
2. Huu ni msimu gani? Jibu si sahihi | Jibu sahihi
3. Huu ni mwezi gani? Jibu si sahihi | Jibu sahihi
4. Leo ni tarehe ngapi? Jibu si sahihi | Jibu sahihi
5. Leo ni siku gani ya juma? Jibu si sahihi | Jibu sahihi

**F2**.

1. Tupo kwenye nchi gani? Jibu si sahihi | Jibu sahihi
2. Tupo kwenye jimbo gani? Jibu si sahihi | Jibu sahihi
3. Tupo kwenye jiji/mji gani? Jibu si sahihi | Jibu sahihi
4. (KAMA NI NYUMBANI), Ipi anuani ya mtaa ya nyumba hii?(KAMA NI KITUO) nini jina la jengo hili? Jibu si sahihi |Jibu sahihi
5. [KAMA NI NYUMBANI], Tupo katika chumba kipi? [HOSPITALINI] Tupo kwenye hodi gani? Jibu si sahihi | Jibu sahihi

**F3**. *[SEMA]: "Nitakutajia majina matatu ya vitu. Nikishamaliza, Nitahitaji wewe uyarudie. Kumbuka ni vitu gani maana nitarudia kukuuliza tena uvitaje ndani ya dakika chache.*

[TAMKA MANENO YAFUATAYO POLEPOLE KWA UMBALI WA SEKUNDE-1]

*“MPIRA,GARI,BINADAMU"* Hakuna sahihi | 1 Sahihi | 2 Sahihi | 3 Sahihi

**F4**. Tahajia (Spell) neno ‘DUNIA’. Sasa litahajie (spell) kinyume:

Hakuna herufi sahihi | 1 Herufi sahihi| 2 Herufi sahihi | 3 Herufi sahihi | 4 Herufi sahihi | 5 Herufi sahihi

**F5**. Sasa vipi vitu vitatu ambavyo nilikuambia uvikumbuke?

Hakuna sahihi | 1 Sahihi | 2 Sahihi | 3 Sahihi

**F6**.

1. ONESHA saa ya mkononi: ULIZA: "Hii inaitwaje?" Jibu si sahihi | Jibu sahihi
2. ONESHA penseli: ULIZA "Hii inaitwaje?" Jibu si sahihi | Jibu sahihi

**F7**. *[SEMA]: “Ningependa urudie msemo huu nikishamaliza kutamka:* 'No ifs, ands or buts.'“

Jibu si sahihi | Jibu sahihi

**F8**. *[SEMA]: "SOMA MANENO KATIKA KURASA NA KISHA FANYA LINAVYOSEMA." [Mpe mshiriki kipande cha karatasi na 'Funga macho yako' ndani yake. Kama wahojiwa anasoma na hawajafunga macho yao, rudia mpaka mara tatu. Weka alama pale tu wahojiwa wamefunga macho yao].*

Hawajafunga macho yao | Wamefunga macho yao

**F9**. MSHIKISHE mhojiwa penseli na karatasi. *[SEMA]: "Andika sentensi yoyote iliyokamilika kwenye kipande cha karatasi."* (Nukuu: Sentensi lazima ilete maana. Achanana na kosa lolote la kispelling.)

Jibu si sahihi | Jibu sahihi

**F10**. WEKA mchoro, Futa na uchore mbele ya mhojiwa. *[SEMA]: "Tafadhali nakili mchoro huu".* [Ruhusu marudio mengi.Subiri mpaka mhojiwa amalize kisha ichukuwe tena.weka maksi kwenye mchoro ambao umenakiliwa sahihi wenye umbo lenye pande-4 kati ya maumbo mawili yenye umbo la pande-5.]

Mchoro si sahihi | Mchoro sahihi

**F11**. MUULIZE mhojiwa kama anatumia mkono wa kulia ama wa kushoto. Chukuwa kipande cha karatasi na ukishikilie mbele ya mhojiwa. *[SEMA]:* *"Chukuwa karatasi hii kwa mkono wako wa kulia/kushoto (Ule ambao hajazoea), Kunja karatasi nusu kwa mara moja kwa kutumia mikono yote na iweke karatasi chini sakafuni.* [Weka maksi 1 kwa kila maelezo yaliyofanyika kwa usahihi.]

0 Karatasi imechukuliwa kwa usahihi mkononi | 1 Imekunjwa nusu | 2 Imewekwa juu ya sakafu

#### Handout for Questionnaire

SWALI **F8**:

FUNGA MACHO YAKO.

SWALI **F9**:

SWALI **F10**:


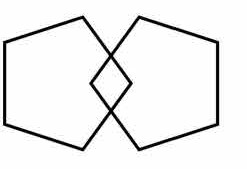

Supplement: Supplementary file 1 — Tanzanian Swahili version of MMSE. (DOCX 25 kb) [file 12883_2019_1283_MOESM1_ESM.docx]
