# Supplementary material for: Cross-cultural adaptation and psychometric properties of the MMSE and MoCA questionnaires in Tanzanian Swahili for a traumatic brain injury population
Source: BMC Neurol. 2019 Apr 8;19:57. doi: 10.1186/s12883-019-1283-9 (PMC6454609; doi:10.1186/s12883-019-1283-9)
Supplement: Supplementary file 2 — Tanzanian Swahili version of MoCA. (DOCX 130 kb) [file 12883_2019_1283_MOESM2_ESM.docx]

**SWAHILI TRANSLATION**

**Montreal Cognitive Assessment (MoCA)**

**KUPIMA MOCA (Montreal Cognitive Assessment)**

**F12.** [Hand the patient the visuospatial test and a pencil/Mpe mgonjwa jaribio la kuona na penseli.]

Hakuna pointi | Pointi 1 | Pointi 2 | Pointi 3 | Pointi 4| Pointi 5

**F13**. Tafdhali nitajie jina la mnyama huyu:

0 Sahihi| 1 Sahihi | 2 Sahihi | 3 Sahihi

KUMBUKUMBU: Soma horodha ya maneno. Subjects must repeat them. Fanya majaribio mawili Hata Kama jaribio la kwanza limefanikiwa.

“USO, NGUO, KANISA, KILA SIKU, NYEKUNDU”

**F14**. Soma horodha ya namba (namba 1 Kwa sekunde.) 2 1 8 5 4. Tafdhali zirudie namba hizi

Jibu si sahihi | Jibu sahihi

**F15**. Soma horodha ya namba (namba 1 Kwa sekunde) 7 4 2. Tafdhali zirudie namba hizi kinyume:

Jibu si sahihi | Jibu sahihi

**F16**. *[SEMA]: "Nitakapokuwa nikisoma horodha hii ya herufi, Tafadhali gusa herufi A."*

"F B A C M N A A J K L B A F A K D E A AA J A M O F A A B "

Jibu si sahihi | Jibu sahihi

**F17**. Unaweza kuanzia kutoka 100 na utoe 7 na uendelee kutoa 7, unapata ngapi? (93, 86, 79, 72, 65)

0 Sahihi| 1 Sahihi | 2 Sahihi | 3 Sahihi

**F18**.

1. Rudia “Tu kwamba John ni mmoja/pekee wa kusaidia leo."

Rudia "Paka huwa anajificha chini ya Kochi wakati mbwa wanapokuwa chumbani."

0 Sahihi | 1 Sahihi | 2 Sahihi

1. Taja maneno mengi uwezavyo ndani ya dakika moja ambayo yanaanzia na herufi “F”.

Maneno 10 au chini | Maneno 11 Au zaidi.

**F19**. Taja mfanano kati ya vitu hivi (ie Ndizi, Chungwa = Tunda) “Treni, Baiskeli" "Saa, rula"

0 Sahihi | 1 Sahihi | 2 Sahihi

**F20**. *[SEMA]: “Unaweza kukumbuka maneno ambayo nilikuambia uyakumbuke*?" (Uso, Nguo, Kanisa, Ua, Nyekundu)

0 Sahihi | 1 Sahihi| 2 Sahihi| 3 Sahihi | 4 Sahihi | 5 Sahihi

#### Handout for Questionnaire

SWALI **F12, F13**:

11:10 (Saa tano na dakika kumi)
