# Supplementary material for: Cross-cultural adaptation and psychometric properties of the MMSE and MoCA questionnaires in Tanzanian Swahili for a traumatic brain injury population
Source: BMC Neurol. 2019 Apr 8;19:57. doi: 10.1186/s12883-019-1283-9 (PMC6454609; doi:10.1186/s12883-019-1283-9)
Supplement: Supplementary file 3 — English version of MMSE. (DOCX 21 kb) [file 12883_2019_1283_MOESM3_ESM.docx]

**ENGLISH**

**Mini-Mental State Exam (MMSE)**

*In the next section, I will ask questions and have you do simple things to check how your brain is functioning. Sometimes it is hard, don't worry. Answer the questions the best you can. You have 10 seconds for most questions. Interviewer, after asking questions, check if the patient has responded to the question correctly.*

F1a. What year is this?                                             Incorrect response  | Correct response

F1b. Which season is this?                                       Incorrect response  | Correct response

F1c. What month is this?                                     Incorrect response  | Correct response

F1d. What is today's date?                                  Incorrect response  | Correct response

F1e. What is the day of the week?                                   Incorrect response  | Correct response

F2a. What country are we in?                       Incorrect response  | Correct response

F2b. What province are we in?                     Incorrect response  | Correct response

F2c. What city/town are we in?                        Incorrect response  | Correct response

F2d. [IF IN HOME], What is the street address of this house?

[IF IN FACILITY] What is the name of this building? Incorrect response  | Correct response

F2e. [If in home], What room are we in?

[IF IN FACILITY] What floor are we on? Incorrect response  | Correct response

F3. [SAY]: "*I am going to name three objects. When I am finished, I want you to repeat them. Remember what they are because I am going to ask you to name them again in a few minutes.*

[SAY THE FOLLOWING WORDS SLOWLY AT 1-SECOND INTERVALS]

"*BALL, CAR, MAN*"          None correct | 1 Correct | 2 Correct | 3 Correct

F4. Spell the word ‘WORLD’. Now spell it backwards

No letters correct | 1 correct | 2 correct | 3 correct | 4 correct | 5 correct

F5. Now what were the three objects that I asked you to remember?

None correct | 1 Correct | 2 Correct |  3 Correct

F6a. SHOW wristwatch:  ASK: "What is this called?"            Incorrect response  | Correct response

F6b. SHOW pencil: ASK "What is this called?"                               Incorrect response  | Correct response

F7. SAY: I would like you to repeat this phrase after me: 'No ifs, ands or buts.'

Incorrect response  | Correct response

F8. SAY: "*READ THE WORDS ON THE PAGE AND THEN DO WHAT IT SAYS*." [Hand the person the sheet with 'close your eyes' on it. If the subject reads and does not close their eyes, repeat up to three times. Score only if the subject closes their eyes.                        Doesn't close their eyes | Closes their eyes

F9. HAND the person a pencil and paper. SAY" *Write any complete sentence on that piece of paper*." (Note: The sentence must make sense. Ignore any spelling errors. )                                                                             Incorrect response  | Correct response

F10. PLACE design, eraser and pencil in front of the person. SAY: "Copy this design please".  [Allow multiple tries. Wait until person is finished and hands it back. Score only for correctly copied diagram with a 4-sided figure between two 5-sided figures.                                                                                                                     Incorrect design |  Correct design


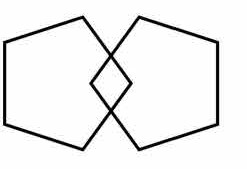


F11. ASK the person if he is right- or left-handed. Take a piece of paper and hold it up in front of the person. SAY "*Take this paper in your right/left hand (whichever is non-dominant), fold the paper in half once with both hands and put the paper down on the floor.* [Score 1 point for each instruction executed correctly.]

Takes paper correctly in hand | Folds it in half | Puts it on the floor
