# Supplementary material for: Cross-cultural adaptation and psychometric properties of the MMSE and MoCA questionnaires in Tanzanian Swahili for a traumatic brain injury population
Source: BMC Neurol. 2019 Apr 8;19:57. doi: 10.1186/s12883-019-1283-9 (PMC6454609; doi:10.1186/s12883-019-1283-9)
Supplement: Supplementary file 4 — English version of MoCA. (DOCX 130 kb) [file 12883_2019_1283_MOESM4_ESM.docx]

**ENGLISH**

**Montreal Cognitive Assessment (MoCA)**

**KUPIMA MOCA (Montreal Cognitive Assessment)**

**F12.** [Hand the patient the visuospatial test and a pencil]

No points | 1 Point |  2 Point | 3 Points | 4 Points |  5 Points

**F13**. SAY "Please name these animals"

0 correct |  1 correct |  2 correct | 3 correct

MEMORY: Read list of words.  Subjects must repeat them. Do 2 trials, even if 1st trial is successful.

“FACE, CLOTHES, CHURCH, EVERYDAY, RED”

**F14**. Read list of digits (1 digit/sec.) 2 1 8 5 4  Please repeat these digits

Incorrect response  | Correct response

**F15**. Read list of digits (1 digit/sec) 7 4 2  Please repeat these digits backwards.

Incorrect response  | Correct response

**F16**. SAY: "As I read this list of letters, please tap at the letter A."

"F B A C M N A A J K L B A F A K D E A AA J A M O F A A B "

Incorrect response  | Correct response

**F17**. Can you start from 100 and subtract 7 and keep substracting 7, what do you get? (93, 86, 79, 72, 65)

0 correct |  1 correct | 2 correct |  3 correct

**F18**. Repeat "I only know that John is the one to help today."

Repeat "The cat always hid under the couch when the dogs were in the room."

0 correct |  1 correct | 2 correct

Name as many words as you can in one minute that start with the letter “F”.

10 or less  | 11 or more words

**F19**. Name the similarity between items (ie banana, orange = fruit)  "Train, bicycle" "Watch, ruler"

0 correct |  1 correct | 2 correct

**F20**. Say "Can you recall the words that I told to you remember?" (Face, Clothes, Church, Everyday, Red)

0 correct |  1 correct | 2 correct |  3 correct | 4 correct | 5 correct

#### Handout for Questionnaire

SWALI **F12, F13**:
